# Supplementary material for: Macropinocytosis confers resistance to therapies targeting cancer anabolism
Source: Nat Commun. 2020 Feb 28;11:1121. doi: 10.1038/s41467-020-14928-3 (PMC7048872; doi:10.1038/s41467-020-14928-3)
Supplement: Supplementary file 1 — Supplementary Information [file 41467_2020_14928_MOESM1_ESM.pdf]

Macropinocytosis confers resistance to therapies targeting cancer anabolism

V. Jayashankar and A.L. Edinger

Supplementary Figures 1-8 with legends

Supplementary Fig. 1 - Related to Fig. 1

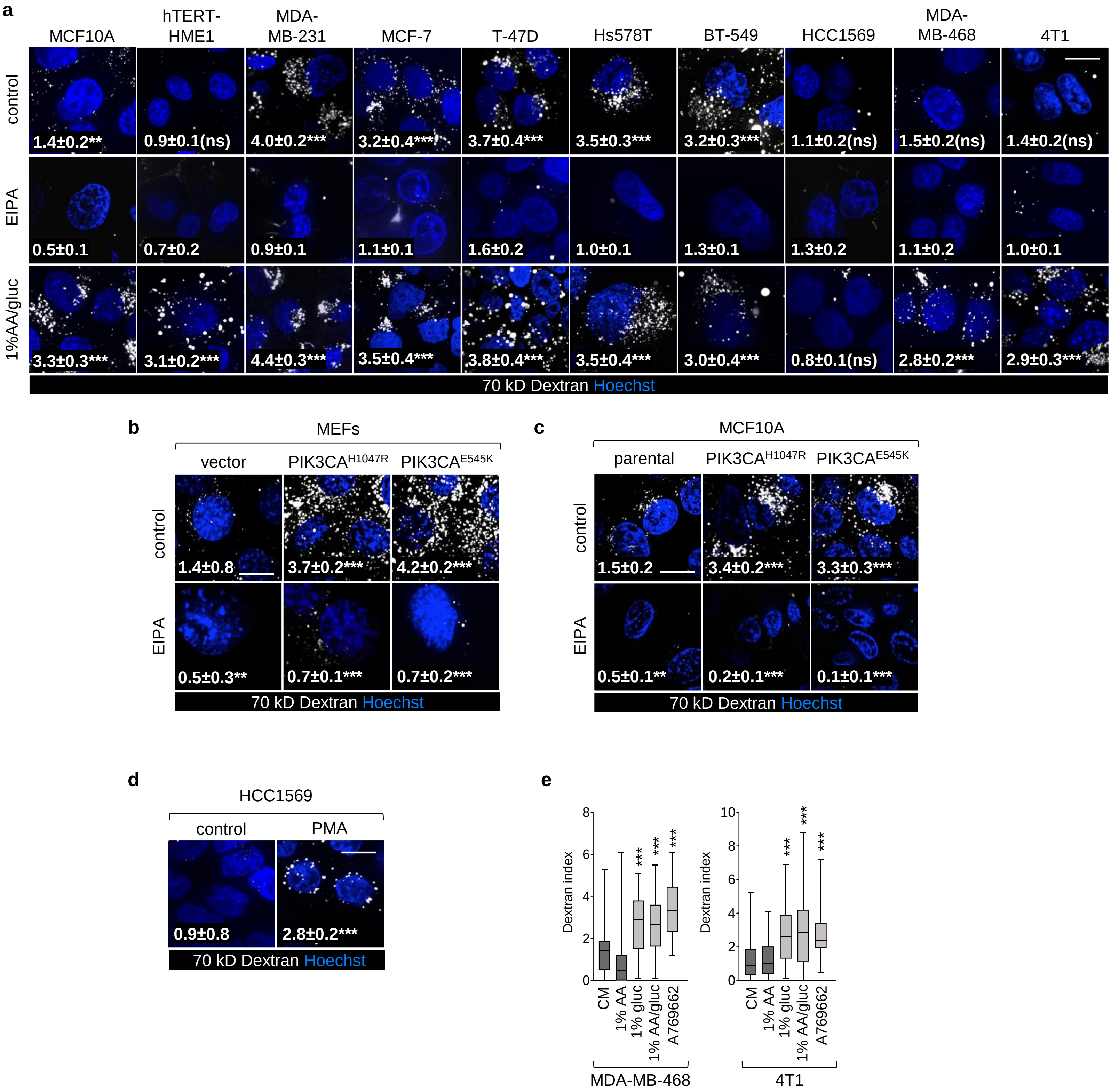

Supplementary Fig. 1: Cells lines with activating mutations in KRAS or the PI3K pathway exhibit macropinocytosis. a 70 kD dextran uptake in complete medium (CM) or 1% AA/glucose medium ± EIPA (50 µM) in the indicated breast cancer cell lines. Representative images for Fig. 1a. Statistics compare ± EIPA. Using an unpaired, two-tailed t-test**,** ***, $P \leq 0.001$; ns, not significant, $P > 0.05$. Forty cells were evaluated from three independent experiments except for MCF10-A and hTERT-HME1 cells where two independent experiments were performed. **b** Dextran uptake in CM ± EIPA (50 µM) in MEFs expressing empty vector, $PIK3CA^{H1047R}$, or $PIK3CA^{E545K}$. **c** Dextran uptake in CM ± EIPA (50 µM) in parental MCF10A cells or in $PIK3CA^{H1047R}$ or ^{E545K} knock-in MCF10A cells. In **b,c,** statistics compare dextran index in control cells and with cells PIK3CA mutations (top row) or ± EIPA (bottom row). Using a one-way ANOVA with Dunnett's correction (top row) or an unpaired, two-tailed t-test (bottom row), **, $P \leq 0.01$ and ***, $P \leq 0.001$; 50 cells were evaluated from two biological replicates. **d** Dextran uptake in HCC1569 cells ± PMA (250 nM). Statistics compare ± PMA; n=30 from one experiment. Using an unpaired, two tailed t-test, ***, P ≤ 0.001. **e** Dextran index in MDA-MB-468 or 4T1 cells maintained in CM, 1% AA, 1% gluc, 1% AA/gluc medium or A769662 (50 µM). Box plots show median at centerline and the 25th to 75th percentile; whiskers represent minimum and maximum values. N=30 cells from two independent experiments. Using a one-way ANOVA with Dunnett's correction, ***, P ≤ 0.001; no asterisk, $P > 0.05$. In **b-d**, mean dextran index ± SEM shown in white. Scale bars, 20 µm.

Supplementary Fig. 2 - Related to Fig. 2

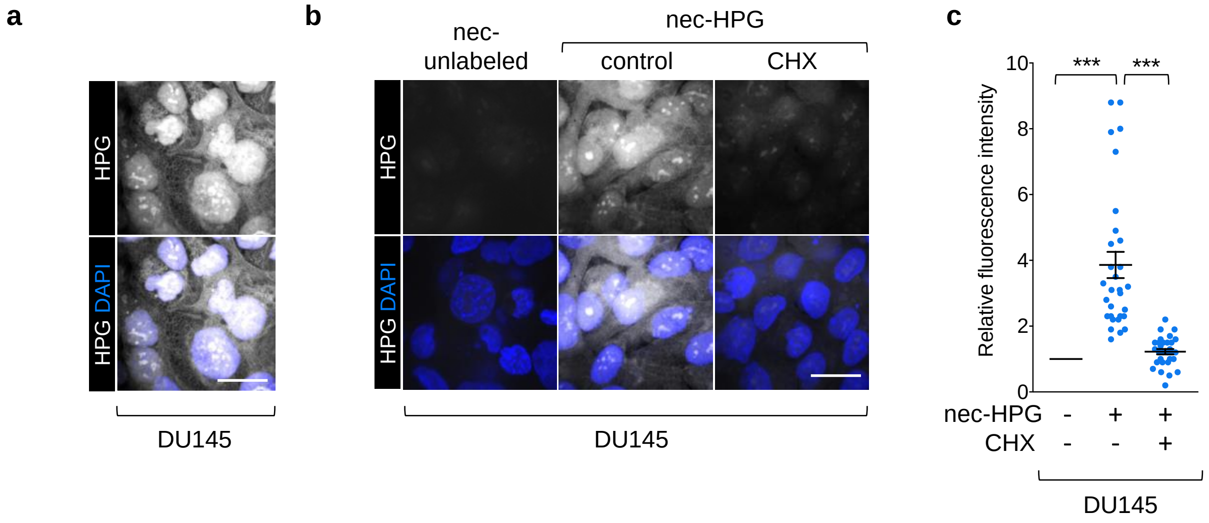

Supplementary Fig. 2: Necrocytosis supports protein synthesis in nutrient-deprived prostate cancer cells. a DU145 cells were labeled with HPG for 24 h in 1% AA. **b** DU145 cells in 1% AA ± cycloheximide (CHX, 50 µg/ml) were supplied with HPG-labeled necrotic cell debris for 24 h. **c** Integrated fluorescence intensity per cell from panel (**b**) normalized to cells fed unlabeled necrotic debris. A total of 50 cells were quantified from 1 experiment; mean ± SEM shown. Using a one-way ANOVA with Tukey's correction, ***, $P \leq 0.001$. Scale bar, 20 µm.

Supplementary Fig. 3 – Related to Fig. 2

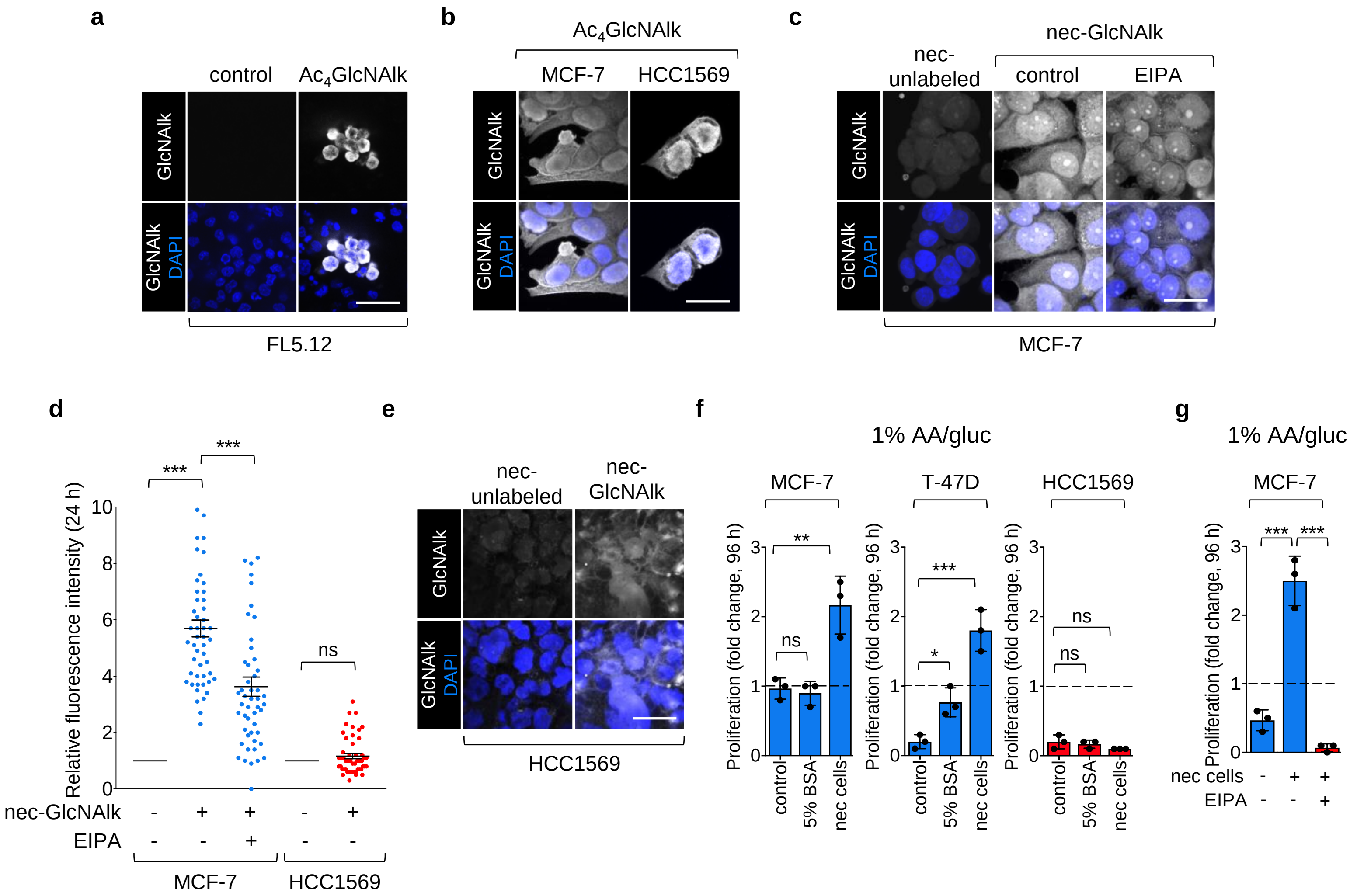

Supplementary Fig. 3: Macropinocytic breast cancer cells scavenge sugars via necrocytosis. a FL5.12 cells labeled with Ac_4GlcNAlk in complete medium for 24 h; GlcNAlk detected with biotin-azide and Alexa488-streptavidin. **b** MCF-7 and HCC1569 cells were labeled as in (**a**) but in 1% AA medium. **c** Macropinocytic MCF-7 cells ± EIPA (50 µM) maintained in 1% AA medium for 24 h with unlabeled or GlcNAlk-labeled necrotic cell debris (nec-GlcNAlk). **d** Integrated fluorescence intensity per cell in (**c**,**e**) normalized to cells fed unlabeled necrotic cell debris. For each condition, 50 cells were examined from two independent experiments. Mean ± SEM shown. Using a one-way ANOVA and Tukey's correction (MCF-7) or an unpaired, two-tailed t-test (HCC1569),***, $P \leq 0.001$; ns, not significant, P > 0.05. **e** As in (**c**), but with non-macropinocytic HCC1569 cells. **f** Proliferation of macropinocytic MCF-7 and T-47D cells or non-macropinocytic HCC1569 cells in 1% AA/gluc medium supplemented with fatty acid-free albumin (5%) or necrotic debris (0.2% protein). **g** MCF-7 cells as in (**f**) but treated with EIPA (25 µM). For **f** and **g**, mean ± SD shown, n=3. Using a one-way ANOVA and Dunnett's (**f**) or Tukey's correction (**g**), *, $P \leq 0.05$; ***, $P \leq 0.001$; ns, not significant, $P > 0.05$. Scale bars, 20 µm.

Supplementary Fig. 4 - Related to Fig. 3

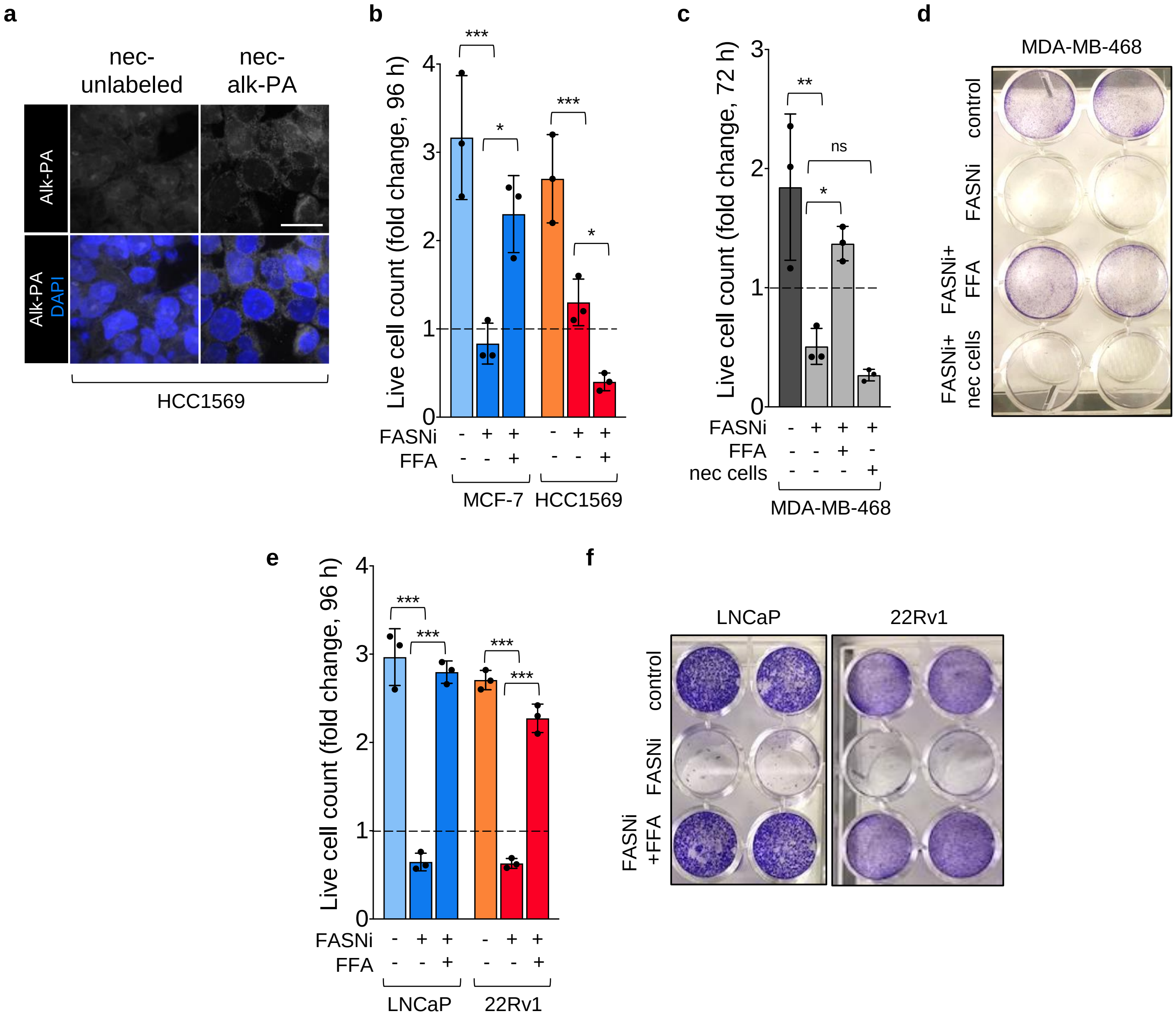

Supplementary Fig. 4: Free fatty acids rescue both non-macropinocytic and macropinocytic cells from fatty acid synthase inhibition. a HCC1569 cells supplemented with unlabeled or alk-PA-labeled necrotic cell debris (nec-alk-PA) and stained with streptavidin-Alexa488. Scale bar, 20 µm**. b** Proliferation of macropinocytic MCF-7 or non-macropinocytic HCC1569 cells in complete medium ± FASNi (GSK2194069, 20 µM) ± linoleic and oleic acid supplementation (1 mg/ml). **c** Proliferation of MDA-MB-468 breast cancer cells as in (**b**). MDA-MB-468 cells are not macropinocytic in complete medium. **d** Representative plate for **c** (duplicate wells shown)**. e** As in (**b**), but using macropinocytic (LNCaP) or non-macropinocytic (22Rv1) prostate cancer cells. **f** Representative plate for **e**. In **b**, **c** and **e,** mean ± SD shown, n=3. Using a one-way ANOVA and Tukey's correction, *, $P \leq 0.05$; **, $P \leq 0.01$; ***, $P \leq 0.001$; ns, not significant, $P > 0.05$.

Supplementary Fig. 5 - Related to Fig. 5

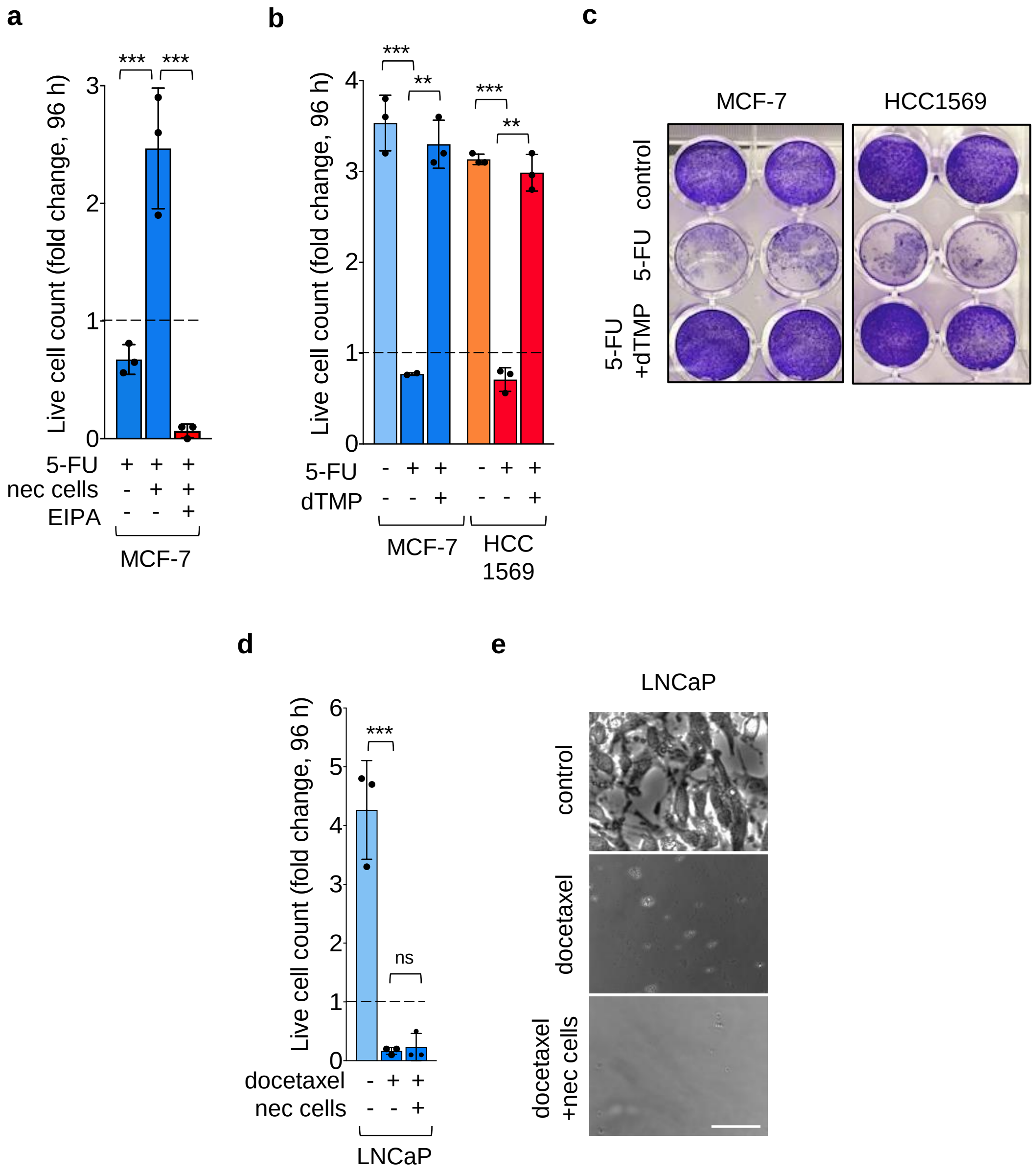

Supplementary Fig. 5: Necrocytosis and nucleotide precursors protect from nucleotide synthesis inhibitors. a Proliferation of macropinocytic MCF-7 ± 5-FU (30 μM) ± necrotic debris (0.2% protein) ± EIPA (25 μM) at 96 h. **b** Proliferation of macropinocytic MCF-7 or non-macropinocytic HCC1569 cells ± 5-FU (30 μM) ± necrotic debris (0.2% protein) ± deoxythymidine monophosphate (dTMP, 1 μM) at 96 h. **c** Representative plate from (**b**). **d** Proliferation of macropinocytic LNCaP cells ± docetaxel (5 μM) ± necrotic debris (0.2% protein) at 96 h. **e** Representative bright field images from (**d**). In **a, b,** and **d,** mean ± SD shown, n=3. Using a one-way ANOVA and Tukey's correction, **, $P \leq 0.01$; ***, $P \leq 0.001$; ns, not significant, $P > 0.05$. Scale bar, 10 μm

Supplementary Fig. 6 - Related to Fig. 6

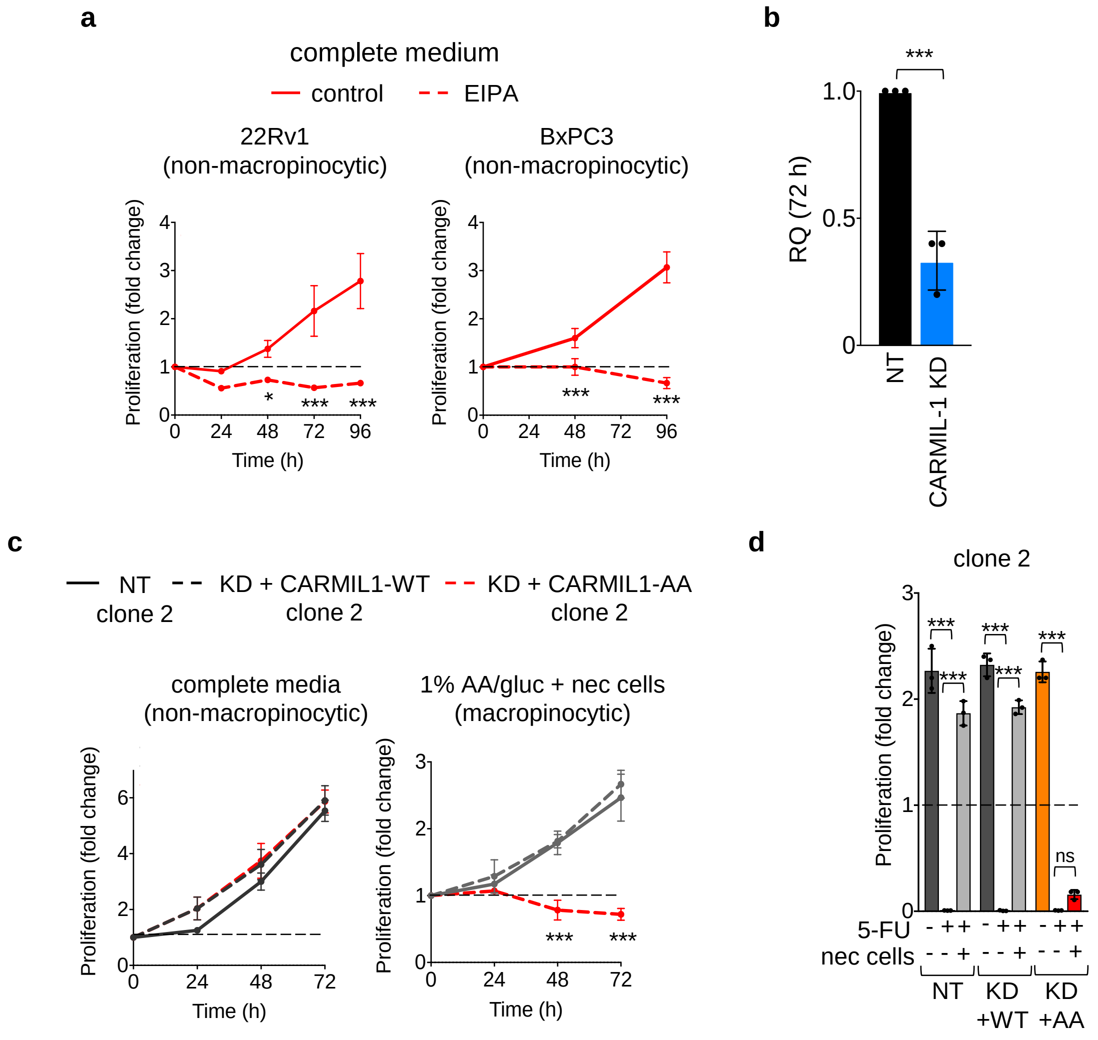

Supplementary Fig. 6: CARMIL1-AA supports normal proliferation, but not macropinocytosis. a Proliferation of non-macropinocytic prostate cancer (22Rv1) or pancreas cancer (BxPC3) cells ± EIPA (25 μM). Using unpaired two-tailed t-tests at each time point, *, *P* ≤ 0.05; ***, *P* ≤ 0.001; no asterisk, *P* > 0.05; mean ± SEM. **b** mRNA levels 72 h post shRNA-mediated knockdown of CARMIL-1 in 4T1 cells, normalized to GAPDH levels. Mean ± SD shown. Using an unpaired, two-tailed t-test, ***, P ≤ 0.001. **c** Proliferation of second clones of contextually macropinocytic 4T1 cells expressing a non-targeting (NT) shRNA or CARMIL1 shRNA (KD) and reconstituted with shRNA-resistant CARMIL1-WT or CARMIL1-AA cDNAs. Assay performed in complete medium or in 1% AA/glucose medium supplemented with necrotic debris (0.2% protein). Using a one-way ANOVA at each time point and Dunnett's correction, ***, *P* ≤ 0.001; no asterisk, *P* > 0.05; mean ± SEM. **d** As in (**c**), but cells were maintained in complete medium ± 5-FU (1 μM) ± necrotic debris (0.2% protein) and proliferation measured at 72 h. Mean ± SEM. Using a one-way ANOVA and Tukey's correction, ***, *P* ≤ 0.001; ns, not significant, *P* > 0.05. For **a-d,** results are derived from three independent experiments.

Supplementary Fig. 7 - Related to Fig. 7

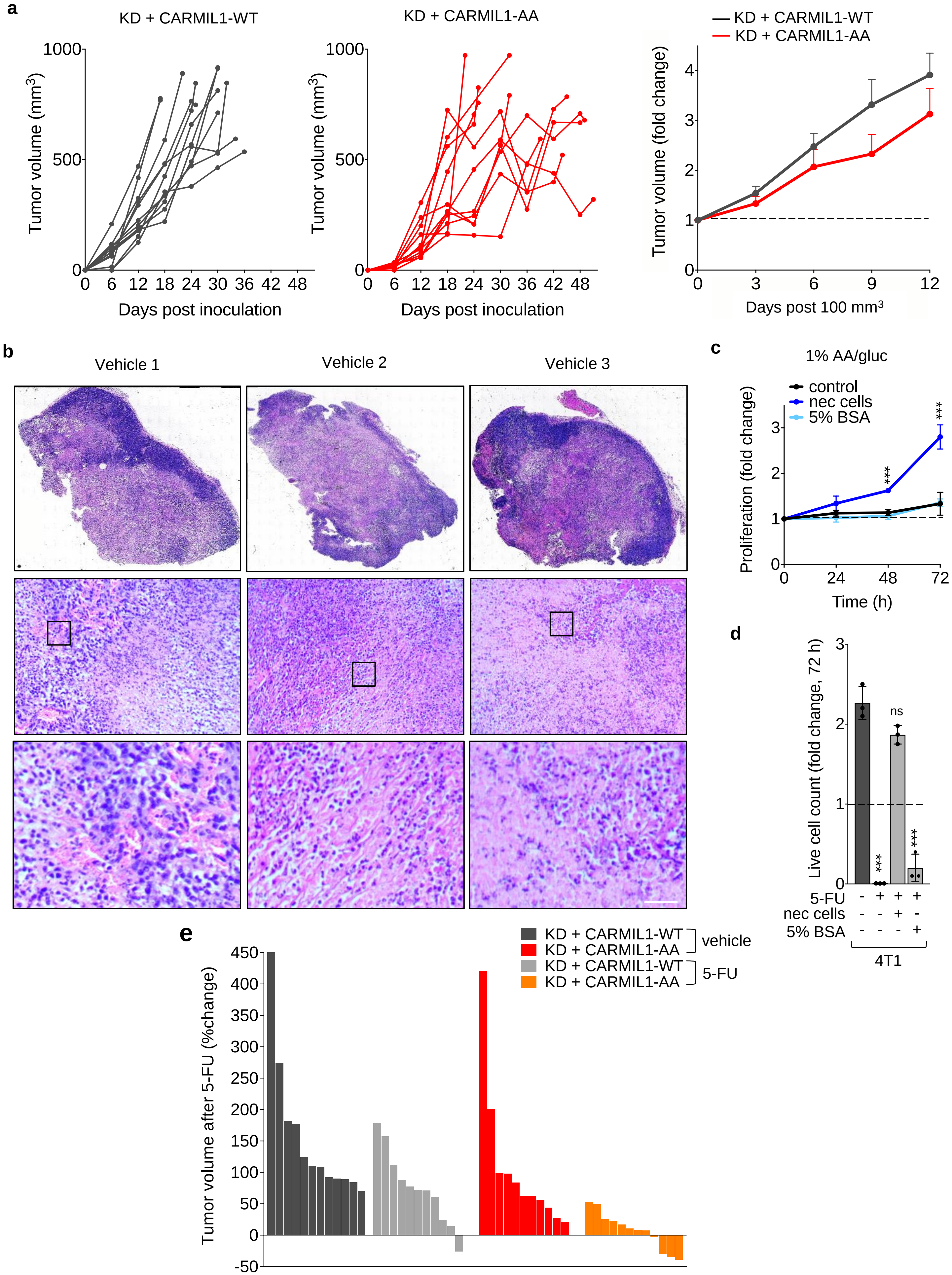

Supplementary Fig. 7: Necrocytosis fuels 5-FU resistance in vivo. a Volume of individual CARMIL1-WT (n=12) or CARMIL1-AA (n=11) tumors. Average growth ± SEM (right panel). Using unpaired, two-tailed t-tests, the difference in average tumor volume (right panel) was not significant at any time point (P > 0.05). **b** H&E staining of three, representative CARMIL1-WT tumors. Tumors estimated to be 70-80% necrotic. Pairwise stitching of 20 X 20 individual frames obtained at a 10X magnification was performed using Zen 2.3 software to produce images of the entire tumor section (top row). Images were also taken at 10X magnification (middle row); a digitally zoomed image is also shown for each tumor (bottom row). Scale bar, 10 µm **c** Proliferation of 4T1 cells maintained in 1% AA/gluc + necrotic debris (0.2% protein) or 5% BSA. Mean ± SD shown, n=3. Using a one-way ANOVA at each time point and Dunnett's correction, ***, *P* ≤ 0.001; no asterisk, *P* > 0.05. **d** Proliferation of 4T1 cells maintained in complete medium ± 5-FU (1 µM) ± necrotic debris (0.2% protein) or BSA (5%) at 72 h. Mean ± SD shown, n=3. Using a one-way ANOVA and Dunnett's correction, ***, *P* ≤ 0.001; ns, not significant, *P* > 0.05. **e** Change in tumor volume after 6 days of treatment with vehicle or 5-FU.

Supplementary Fig. 8

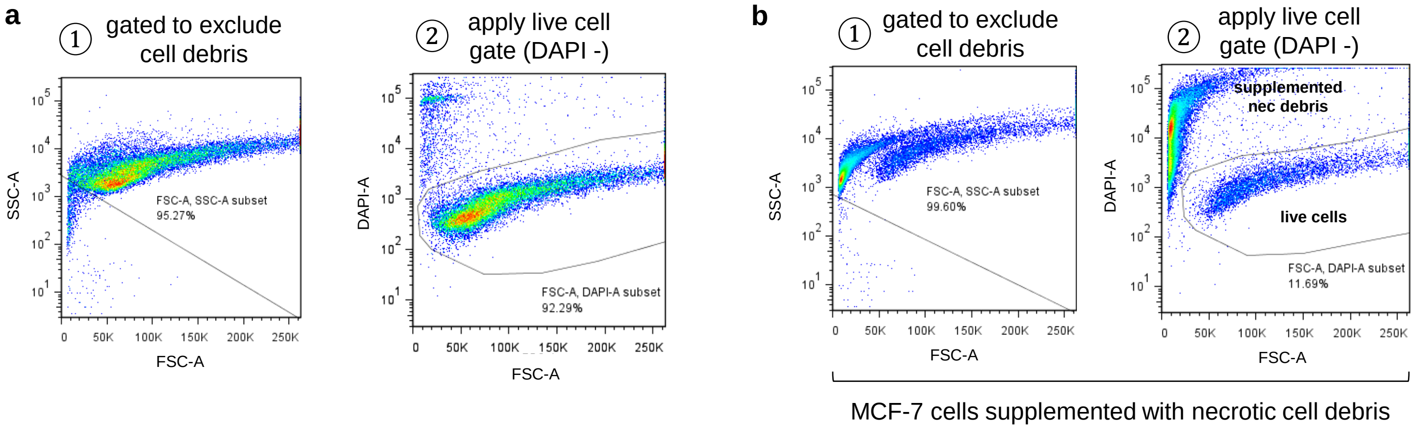

Supplementary Fig. 8: Flow cytometry gating strategy for proliferation assays. a Cell debris was excluded using a FSC/SSC dot plot. DAPI negative events were considered live cells. **b** A similar strategy was used where cells were supplemented with necrotic debris. The number of live cells collected in 30 sec was used to monitor proliferation.
