# Supplementary material for: Macropinocytosis confers resistance to therapies targeting cancer anabolism
Source: Nat Commun. 2020 Feb 28;11:1121. doi: 10.1038/s41467-020-14928-3 (PMC7048872; doi:10.1038/s41467-020-14928-3)
Supplement: Supplementary file 3 — Reporting Summary [file 41467_2020_14928_MOESM3_ESM.pdf]

nature research

Corresponding author(s): Aimee L. Edinger

Last updated by author(s): Jan 20, 2020

# Reporting Summary

Nature Research wishes to improve the reproducibility of the work that we publish. This form provides structure for consistency and transparency in reporting. For further information on Nature Research policies, seeAuthors & Referees and theEditorial Policy Checklist .

## Statistics

For all statistical analyses, confirm that the following items are present in the figure legend, table legend, main text, or Methods section.

| n/a | Confirmed | |
|---|---|---|
| ☐ | ☒ | The exact sample size (*n*) for each experimental group/condition, given as a discrete number and unit of measurement |
| ☐ | ☒ | A statement on whether measurements were taken from distinct samples or whether the same sample was measured repeatedly |
| ☐ | ☒ | The statistical test(s) used AND whether they are one- or two-sided<br>*Only common tests should be described solely by name; describe more complex techniques in the Methods section.* |
| ☐ | ☒ | A description of all covariates tested |
| ☐ | ☒ | A description of any assumptions or corrections, such as tests of normality and adjustment for multiple comparisons |
| ☐ | ☒ | A full description of the statistical parameters including central tendency (e.g. means) or other basic estimates (e.g. regression coefficient) AND variation (e.g. standard deviation) or associated estimates of uncertainty (e.g. confidence intervals) |
| ☐ | ☒ | For null hypothesis testing, the test statistic (e.g. *F*, *t*, *r*) with confidence intervals, effect sizes, degrees of freedom and *P* value noted<br>*Give P values as exact values whenever suitable.* |
| ☒ | ☐ | For Bayesian analysis, information on the choice of priors and Markov chain Monte Carlo settings |
| ☒ | ☐ | For hierarchical and complex designs, identification of the appropriate level for tests and full reporting of outcomes |
| ☒ | ☐ | Estimates of effect sizes (e.g. Cohen's *d*, Pearson's *r*), indicating how they were calculated |

*Our web collection on statistics for biologists contains articles on many of the points above.*

## Software and code

Policy information about availability of computer code

| | |
|---|---|
| Data collection | Micro-manager, version 2.0; open source microscopy software or Zeiss ZEN version 2.3 imaging software was used to acquire all images presented in the manuscript. BD FACSDIVA V5.0.3 was used to collect flow cytometry data. Microplate software Gen5 version 2.09 was used to measure absorbance from crystal violet. |
| Data analysis | Image J, version 2; open source image processing software. GraphPad Prism, version 7; Statistical analysis and graphing software. Flow cytometry data was analyzed using BD FACSDIVA version 5.0.3. |

For manuscripts utilizing custom algorithms or software that are central to the research but not yet described in published literature, software must be made available to editors/reviewers. We strongly encourage code deposition in a community repository (e.g. GitHub). See the Nature Research guidelines for submitting code & software for further information.

## Data

Policy information about availability of data

All manuscripts must include a data availability statement. This statement should provide the following information, where applicable:

- Accession codes, unique identifiers, or web links for publicly available datasets
- A list of figures that have associated raw data
- A description of any restrictions on data availability

Data supporting the findings of this study are available within the paper, its supplementary information files and the source data file. A reporting summary for this article is available as a supplementary information file. All microscopy data has been deposited in the image data resource (IDR) public repository All other data can be made available from the authors upon request

# Field-specific reporting

Please select the one below that is the best fit for your research. If you are not sure, read the appropriate sections before making your selection.

- [x] Life sciences
- [ ] Behavioural & social sciences
- [ ] Ecological, evolutionary & environmental sciences

For a reference copy of the document with all sections, see nature.com/documents/nr-reporting-summary-flat.pdf

# Life sciences study design

All studies must disclose on these points even when the disclosure is negative.

| | |
|---|---|
| Sample size | For all quantitative microscopy data due to large effect size, the use of stringent negative and positive controls and high reproducibility, a sample size of 30-100 cells were evaluated from 2-4 biological replicates. Sample size for animal studies was determined based on power analysis using data from pilot studies. In rare cases (Fig. 1f, Fig 2d,e supplemental Fig. 1d and and supplemental Fig. 2) data from 30-100 cells was analyzed from one biological replicate. These instances reflect the use of a common positive control (Fig. S1d), the result has been previously reported by our group or others (Fig. 1f), or the results were consistent with complementary experiments in this manuscript or other publications (Fig 2d,e, Fig. S1d and Fig S2). |
| Data exclusions | For all microscopy data, mitotic cells were excluded from analysis. |
| Replication | All experiments were replicated as indicated in the figure legends and methods. All replicated data were reproducible. |
| Randomization | For all vivo experiments, mice were randomly distributed to vehicle or 5-FU treatment groups such that mean tumor volume in both groups was similar. An independent researcher supervised each randomization. For in vitro experiments, wells were randomly assigned as control or treated samples. |
| Blinding | Researchers were not blinded because it was not feasible to conduct truly blinded experiments with the laboratory staff available. Moreover, as effects were unambiguous, it would be difficult to maintain blinding. For in vivo tumor studies, an independent researcher validated caliper measurements approximately weekly. In addition, independent researchers were routinely asked to evaluate unlabeled microscopy images to confirm conclusions drawn from quantification performed by the primary experimenter. Microscopy was quantified using ImageJ in an unbiased manner (per field or per cell measurements) to avoid contamination by experimenter bias. Together, these actions reduce the risk that bias affected the conclusions drawn this manuscript. |

# Reporting for specific materials, systems and methods

We require information from authors about some types of materials, experimental systems and methods used in many studies. Here, indicate whether each material, system or method listed is relevant to your study. If you are not sure if a list item applies to your research, read the appropriate section before selecting a response.

## Materials & experimental systems

| n/a | Involved in the study | |
|---|---|---|
| x | | Antibodies |
| | x | Eukaryotic cell lines |
| x | | Palaeontology |
| | x | Animals and other organisms |
| x | | Human research participants |
| x | | Clinical data |

## Methods

| n/a | Involved in the study | |
|---|---|---|
| x | | ChIP-seq |
| | x | Flow cytometry |
| x | | MRI-based neuroimaging |

## Eukaryotic cell lines

Policy information about cell lines

| | |
|---|---|
| Cell line source(s) | MDA-MB-231, MDA-MB-468, MCF-7, T-47D, BT-549, Hs578T, HCC1569, hTERT-HME1 PANC-1, BxPC3 and LNCaP, cells were obtained from the ATCC. 4T1 murine mammary carcinoma cell line was obtained from Jennifer Prescher (UCI). 22Rv1 cells were obtained from Ionis Pharmaceuticals. MCF10A cells were supplied by Ben Ho Park (Johns Hopkins School of Medicine). All other cell lines were obtained from the ATCC. 4T1 and MCF10A were obtained from the ATCC by the Prescher or Park laboratories, respectively. |
| Authentication | Loss of PTEN (BT-549, MDA-MB-468 and HCC1569) were verified via western blotting for PTEN expression. Other cell lines were not authenticated. |
| Mycoplasma contamination | 4T1 cells were cured of Mycoplasma by culturing in ciprofloxacin for 8 wks. Mycoplasma clearance was confirmed by PCR. All other cell lines tested negative for Mycoplasma. |
| Commonly misidentified lines (See ICLAC register) | These cell lines were not used. |

## Animals and other organisms

Policy information about studies involving animals; ARRIVE guidelines recommended for reporting animal research

| | |
|---|---|
| Laboratory animals | 5-6 wk old female BALB/c mice obtained from Jackson Laboratory (stock no. 000651). |
| Wild animals | Wild animals were not used in this study. |
| Field-collected samples | Field-collected samples are not part of this study. |
| Ethics oversight | UC Irvine's IACUC (Institutional Animal Care and Use Committee) |

Note that full information on the approval of the study protocol must also be provided in the manuscript.

## Flow Cytometry

### Plots

Confirm that:

- [x] The axis labels state the marker and fluorochrome used (e.g. CD4-FITC).
- [x] The axis scales are clearly visible. Include numbers along axes only for bottom left plot of group (a 'group' is an analysis of identical markers).
- [x] All plots are contour plots with outliers or pseudocolor plots.
- [x] A numerical value for number of cells or percentage (with statistics) is provided.

### Methodology

| | |
|---|---|
| Sample preparation | Cell proliferation was determined by flow cytometry by recording the number of cells that excluded vital dye (DAPI (1 mg/mL) or PI (1 mg/mL)) over a fixed collection interval (30 sec) |
| Instrument | BD LSR II |
| Software | BD FACSDIVA V5.0.3 |
| Cell population abundance | The entire population of cells was analyzed. |
| Gating strategy | Debris was excluded by gating on live, untreated cells in a FSC/SSC plot. To obtain live cell counts, DAPI or propidium iodide positive events were excluded. Gating strategy is illustrate in Supplemental Figure 8. |

- [x] Tick this box to confirm that a figure exemplifying the gating strategy is provided in the Supplementary Information.
